# Auxiliary Medical Decision System for Prostate Cancer Based on Ensemble Method

**DOI:** 10.1155/2020/6509596

**Published:** 2020-05-18

**Authors:** Jia Wu, Qinghe Zhuang, Yanlin Tan

**Affiliations:** ^1^School of Computer Science and Engineering, Central South University, Changsha 410083, China; ^2^“Mobile Health” Ministry of Education-China Mobile Joint Laboratory, Changsha 410083, China; ^3^PET-CT Center, The Second Xiangya Hospital of Central South University, Changsha 410083, China

## Abstract

Prostate cancer (PCa) is one of the main diseases that endanger men's health worldwide. In developing countries, due to the large number of patients and the lack of medical resources, there is a big conflict between doctors and patients. To solve this problem, an auxiliary medical decision system for prostate cancer was constructed. The system used six relevant tumor markers as the input features and employed classical machine learning models (support vector machine and artificial neural network). Stacking method aimed at different ensemble models together was used for the reduction of overfitting. 1,933,535 patient information items had been collected from three first-class hospitals in the past five years to train the model. The result showed that the auxiliary medical system could make use of massive data. Its performance is continuously improved as the amount of data increases. Based on the system and collected data, statistics on the incidence of prostate cancer in the past five years were carried out. In the end, influence of diet habit and genetic inheritance for prostate cancer was analyzed. Results revealed the increasing prevalence of PCa and great negative impact caused by high-fat diet and genetic inheritance.

## 1. Introduction

In 2018, morbidity and mortality of PCa accounted for 13.5% and 6.7%, respectively, in male patients. In 185 countries around the world, PCa has the highest morbidity in 105 countries and the highest mortality in 46 countries [1]. Undoubtedly, PCa has become one of the main threats to men's health worldwide. Countries with high HDI (human development index) usually have high morbidity (68.0/100,000). Although countries with low HDI have relatively low morbidity (14.5/100,000), there is an obvious trend of growth and it increases fast [2]. Take China for example; in 1998, the rough morbidity was 3.25/100,000; however, in 2012, it increased to 8.14/100,000. By 2013, it has become 8.58/100,000 [3, 4]. The high morbidity in developed countries and the increasing incidence rate in developing countries have led to a huge number of prostate cancer patients worldwide.

In developing countries that lack medical resources, many patients cannot receive timely and effective diagnosis and therapy, which will aggregate the conflict between doctors and patients. In China, there are only 2.59 practitioners for every 1000 people [5]. In Beijing, a small number of high-level hospital medical staff members have to provide medical services to more than 20 million people in Beijing, and many cancer patients in other regions also come here for the extraordinary medical treatment. Medical staff members have been overloaded for a long time [6–9]. This will inevitably have an impact on the accuracy of the diagnosis, thus leading to serious consequences.

Other developing countries may face similar problems:

- Due to the large number of patients and less medical resources, it is difficult for patients to get timely and effective diagnosis and treatment
- The long-term workload of doctors reduces the efficiency and accuracy of diagnosis
- Many hospitals have poor medical equipment, which further increases the probability of misdiagnosis
- The per capita income in developing countries is pretty low, and most people cannot afford expensive but accurate diagnostic methods like PET-CT

These problems can be alleviated by building an auxiliary medical decision system. By analyzing a large number of data, the auxiliary medical decision system can learn a diagnostic model. When a new patient comes, it will provide doctors with suggestions relevant to diagnosis or treatment based on the learned model [10, 11]. Combing suggestions from the system and their own knowledge, doctors will give the final conclusion of diagnosis and treatment method. The auxiliary medical decision system can relieve the doctor's burden to some extent, thus alleviating the conflict between doctors and patients [12, 13]. In this work, we constructed an auxiliary medical decision system which can determine whether a patient has prostate cancer, judge the clinical stage, recommend treatment options, and evaluate the effectiveness of treatment options. Given the low income in developing countries, six tumor markers are selected with relatively low testing price and high relevance to PCa as the input features. Classical machine learning techniques and ensemble method are adopted to extract the knowledge inside data and improve performance.

The main contributions and innovations of this research include the following:

- Appropriate features are selected for the construction of medical systems according to national conditions of developing countries
- The constructed auxiliary system can give treatment plan and evaluate its effectiveness
- The use of constructed ensemble method by a secondary learner improved the accuracy
- The system is trained based on a large amount of patient information from three high-level hospitals in China, and some factors affecting PCa via the constructed system are analyzed

The main structure of this article is as follows: the first part describes the background and contribution of the research, the second part introduces related research, the third part is a detailed description to the construction of the model, the fourth part is about the training process and analysis to experiments' results, and the fifth part is the conclusion.

## 2. Related Works

Medical diagnosis of cancer is usually a gradual transition which starts from simple, cheap, and harmless but with low-accuracy methods and ends with expensive and accurate methods. Compared with commonly used CT, MRI, PET-CT, and other methods, detection of tumor markers is a relatively basic and cheap method in the diagnosis of cancer, which makes constructing the auxiliary medical decision system with tumor markers suitable for developing countries with low capital medical expenditure, low medical level, and poor medical facilities. Diagnosing cancer with a single tumor marker usually does not have good sensitivity and specificity [14]. Therefore, many related research studies [14–17] combine different tumor markers or biomarkers to predict some diseases and have acquired good results. Specifically, literature [15] detected serum levels of 17 tumor markers for 145 patients with pancreatic cancer and selected 9 tumor markers by backward elimination selection, scatter plots, and relative operating characteristic analysis. Based on these features, the pancreatic cancer determination system CAMPAS-P was established. The final results showed that the CAMPAS-P system was able to accurately distinguish malignant pancreatic cancer from benign pancreatobiliary disease but performed bad on the diagnosis of the unusual histologic type of pancreatic tumors and various digestive organ malignancies. Literature [17] used serum microRNA biomarkers to predict nonalcoholic fatty liver disease (NAFLD). Among all the selected biomarkers, nine of them were associated with NAFLD severity, and some of them appeared specific to NAFLD. These biomarkers showed good classification performance for nonalcoholic steatohepatitis (NASH). Literature [14] combined growth-related tumor markers and associated tumor markers for the diagnosis of cancer and acquired 80-90% sensitivity, 84-85% specificity, and 83-88% accuracy.

As one of the classic machine learning algorithms, the support vector machine (SVM) [18] shows good performance in many classification problems before the revival of deep learning. It is also widely used in medical diagnosis [19–23]. Literature [19] extracted features from mammograms by Hough transform and classified mammograms by SVM. Its accuracy reached 94% while other machine learning methods like linear discriminant analysis just had 86% accuracy. Literature [20] employed various machine learning techniques for the prediction of breast cancer in Wisconsin Breast Cancer (original) datasets. After considering accuracy, sensitivity, specificity, and precision, SVM got the best results. Literature [21] proposed a classification fuzzy-rough set with the SVM model featured as CA-125 and other amino acids to detect early-stage ovarian cancer. It performed quick learning and had good classification performance.

Ensemble learning technology is also widely used in medical assistant diagnosis. Literature [24] selected demographic, physiological, and vital signs and laboratory tests as features and built different models, finding that the ensemble learning random forest is the most effective in mortality prediction in the early hours of an ICU patient admission. The proposed EMPICU-RF framework based on the ensemble learning random forest outperformed many standard scoring systems in terms of AUROC (area under the curve) and time. Literature [25] proposed a model that combines the physicians' knowledge in the form of a rule-based classifier and supervises learning algorithms to detect asthma control level. Literature [23] constructed two different ensemble models by confidence-weighted voting method and the boosting ensemble technique for the diagnosis of breast cancer. The proposed CWV-BANN-SVM model reached the accuracy of 100%.

## 3. Design of the Auxiliary Medical Decision-Making System

### 3.1. Requirements and Framework of the System

The auxiliary medical decision system is aimed at offering some help to doctors. Its functions cover diagnosing the patients, staging the cancer, recommending the treatment plan, and evaluating the treatment plan. Diagnosing patients is to tell if the tumor is malignant or benign. Staging the cancer is to determine the clinical stage (I, II, III, or IV) for those diagnosed with malignant PCa. The above two requirements can be satisfied by building a classification model using the machine learning method. On the other hand, in order to give a cancer treatment plan and evaluate its efficacy at the same time, the whole problem is considered a regression problem. The system will finally output a value evaluating the malignancy of PCa, abbreviated as EM value. The larger the value, the higher the malignancy. If the value does not decrease after executing a certain treatment plan, it means that the treatment plan is not effective and another treatment plan needs to be selected. Meanwhile, the auxiliary medical decision-making system needs to have good parallelism and be able to process multiple patients' simultaneous diagnosis requests. It is worth noting that after the medical system is invested, the amount of data obtained will gradually increase over time. The decision model will be retrained to further improve the generalization performance.

### 3.2. Design of the Decision Model

#### 3.2.1. Introduction to the Support Vector Machine

The support vector machine can acquire the global optimal solution in high-dimensional problems; thus, it is widely used in many situations [26].

For a linear separable binary classification problem, assume that the input dataset*S* = {*x*_1_, *x*_2_, *x*_3_, ⋯, *x*_*m*_} and the output label *y* = {*y*_1_, *y*_2_, *y*_3_, ⋯, *y*_*m*_}, where *x*_*i*_ is the input vector of the *i*th sample and *y*_*i*_ ∈ {−1, 1} is the corresponding label of *x*_*i*_. SVM aims to find a hyperplane *H* : *W*^T^*x* + *b* = 0 that separates the positive and negative samples and meanwhile maximizes their distance to the hyperplane. The optimizing process can be expressed as follows:

(1) $$\begin{array}{c} \left\{\begin{array}{c} \underset{w,b}{\min}\frac{1}{2{\left\|W\right\|}^{2}},\\ y_{i}\left(W^{\text{T}}x+b\right)\geq 1,\;i=1,2,\cdots ,m. \end{array}\right. \end{array}$$

In order to compute the solution efficiently, the Lagrange multiplier is introduced, and according to the Wolfe duality theory, it is changed into an equivalent dual problem:

(2) $$\begin{array}{c} \text{s}.\text{t}.\overset{m}{\underset{i=1}{\sum }}y_{i}\alpha_{i}=0,\;\alpha_{i}\geq 0,i=1,2,\cdots ,m. \end{array}$$

As for the linear inseparable problem, penalty parameter *C* and slack variable *ξ*_*i*_ are introduced, and the problem is expressed as follows:

(3) $$\begin{array}{c} \begin{array}{c} \underset{\alpha }{\min}\frac{1}{2}\overset{m}{\underset{i=1}{\sum }}\overset{m}{\underset{j=1}{\sum }}y_{i}y_{j}\alpha_{i}\alpha_{j}x_{i}^{\text{T}}x_{j}-\overset{n}{\underset{j=1}{\sum }}\alpha_{j}\\ \text{s}.\text{t}.\;\overset{m}{\underset{j=1}{\sum }}y_{i}\alpha_{i}=0\;0\leq \alpha_{i}\leq C,i=1,2,\cdots ,m, \end{array} \end{array}$$

where *C* represents the interval of two classes, and the final decision function is as follows:

(4) $$\begin{array}{c} y\left(x\right)=\mathrm{sgn}\left(f\left(x\right)\right)=\mathrm{sgn}\left\{\left(\overset{m}{\underset{i=1}{\sum }}\alpha_{i}^{*}y_{i}\left(x_{i}^{\text{T}}\cdot x\right)\right)+b^{*}\right\}. \end{array}$$

For the nonlinear problem, kernel function *φ*(*x*_*i*_) that maps the nonlinear problem in low dimension into a linear problem in high dimension is introduced. The kernel functions defined in the input sample space satisfy the Mercer condition and have the following expression: *K*(*x*_*i*_, *x*_*j*_) = (*φ*(*x*_*i*_), *φ*(*x*_*j*_)) = *φ*(*x*_*i*_)^T^*φ*(*x*_*i*_). Commonly used kernel functions are listed in Table 1.

A typical support vector machine is usually used to deal with binary classification problems. In this medical decision system, SVM is firstly used to classify benign (labeled 1) and malignant (labeled -1) tumors. In order to stage possible malignant tumors, a four-class (I, II, III, or IV) classification task is completed by one-to-one method, which means to train $\left(\begin{array}{c} 4\\ 2 \end{array}\right)$ SVMs simultaneously and integrate the results of each SVM by majority voting. In the training process of one-to-one method, each SVM only needs the data in two classes which will have smaller training cost and keep the generalization performance at the same time.

The selection of the kernel function is one of the main factors that influence the performance of SVM. Commonly used kernel functions include linear function, polynomial function, sigmoid function, and radial basis function. Here, multiple kernel functions are used simultaneously to construct the SVM-based multiclassifiers in case of poor generalization performance due to wrong kernel function selection.

#### 3.2.2. Introduction to the Neural Network

The neural network (NN) is a model with strong fitting ability and is widely used by researchers in various disciplines. It is mainly composed of an input layer, hidden layers, and an output layer. According to the structure of the network, NN can be divided into multiple types: multilayer perceptron (MLP) neural network, radial basis function (RBF) neural network, adaptive resonance theory (ART) neural network, self-organizing map (SOM) neural network, etc. There are two main network models used in this medical decision system, MLP neural network and RBF neural network.

**Figure 1:**
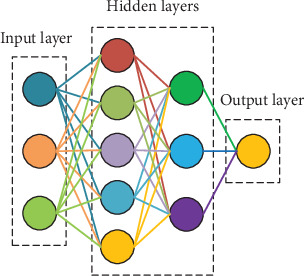
Schematic diagram of MLP.

The MLP neural network contains one input layer, one or more hidden layers, and one output layer. Usually, every two adjacent layers are fully connected as shown in Figure 1.

The activation function of the MLP neural network is usually the rectified linear unit or the ReLU function which can be expressed as *f*(*x*) = max(0, *x*).

**Figure 2:**
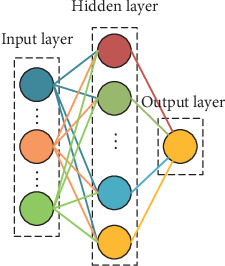
Schematic diagram of the RBF neural network.

The RBF neural network is another widely used neural network [27]. It converges fast and has strong generalization ability. Unlike the MLP neural network, the RBF network contains only one hidden layer and uses radial basis function *f*(*x*, *c*_*i*_) = exp(−*β*_*i*_‖*x* − *c*_*i*_‖^2^) as the activation function, where *c*_*i*_ is the center of the *i*th unit in the hidden layer. The structure of the RBF neural network is shown in Figure 2.

#### 3.2.3. Ensemble Learning

Ensemble learning is a method that integrates many base learners together to improve the overall learning ability. Commonly used ensemble learning methods include boosting, bagging, and stacking. The stacking method can generate all base learners in parallel and uses a secondary learner to integrate the results of the base learners. This integration method has suitable training cost and strong generalization performance. In our medical decision-making system, in order to provide corresponding treatment plan recommendations and evaluate the efficacy of the plan after staging the cancer, the results of the classifier needs to be converted into a regression value. In this case, stacking is a good choice. To make ensemble method really work, base learners need to have some difference. This difference may come from different models, different input datasets, or different input features. Given that SVM and neural networks are less sensitive to the input dataset and our feature set is small, it is not suitable to use different input samples or different input features to improve generalization performance. However, there are many choices in the selection of the kernel function for SVM and structure for neural networks. Therefore, parameter perturbation is taken to enlarge the difference between base learners so that our decision model can be strengthened. Finally, the weights of base learners are learned through exponential linear regression (ELR) to obtain the evaluation of tumor malignancy or the EM value.

### 3.3. Detailed Description of the Medical Decision System

**Figure 3:**
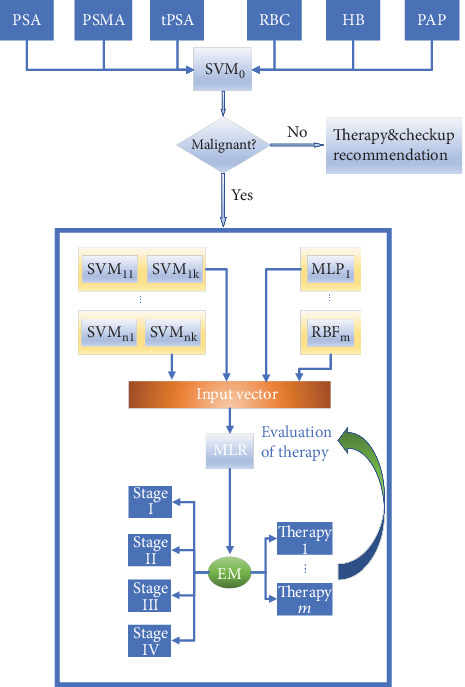
The main flow of the auxiliary medical decision system.

In the proposed medical decision system, six important tumor markers including prostate-specific antigen (PSA), prostate-specific membrane antigen (PSMA), total prostate-specific antigen (tPSA), red blood cell (RBC), hemoglobin (HB), and prostate acid phosphatase (PAP) are chosen for the diagnosis of PCa as the input features and SVM as the diagnosis model. Clinical stage determination of malignant PCa, treatment recommendation, and evaluation are completed by an ensemble model that combines SVM groups for four-class classification and neural networks with different structures.  Figure 3 depicts the main flow of the auxiliary medical system.

First, relevant data from different hospital systems are collected. Then, six important tumor markers' levels are extracted from thousands of information items. After dropping samples with missing or abnormal value, an input vector *x* = (*x*_PSA_, *x*_PSMA_, *x*_tPSA_, *x*_RBC_, *x*_HB_, *x*_PAP_) is formed. Next, it will firstly use SVM to judge if the tumor is malignant. In clinical medicine, the increase in tumor marker level does not mean the development of a malignant tumor for sure. Many benign lesions or inflammations may also lead to an increase in tumor marker level, but the increase is not large. When the system determines that the tumor is benign, recommendations about the next examination and corresponding treatment will be listed.

If the tumor is judged to be malignant, the ensemble model will complete the stage division. The development of the malignant tumor will be divided into four stages: I, II, III, and IV; that is, to say, the system must complete a four-classification task. Since SVM is mainly used at binary classification problems, one-to-one strategy is taken and every six SVM models form a group of SVMs. The output of each group is a voting combination of six binary SVM classifiers in the group, which is represented by a four-dimensional one-hot vector. The differentiation of the SVM classifier is realized by choosing different kernel functions to improve the final performance. More explicitly, SVMs in the same group use the same kernel function, and SVMs in different groups use different kernel functions. Three commonly used kernel functions: linear kernel, polynomial kernel, and Gaussian kernel are chosen to differentiate SVM groups.

While training, parameters in the kernel function and penalty parameters of each binary SVM are adjusted to reduce the generalization error below the threshold *ε*. In order to further reduce the risk, the widely used MLP neural network and RBF neural network are added into the system. Because 6 input features are selected and samples are classified into four classes, the input and output layers of the MLP and RBF networks are 6 units and 4 units, respectively. Three group MLP neural networks with different structures are selected: 6-9-7-4, 6-10-7-5-4, and 6-7-5-4 (these numbers follow the order of unit numbers in each layer). The ReLU function is used as the activation function in MLP neural networks. Similarly, three RBF networks with different structures are used. The hidden unit numbers in three networks are set as 10, 14, and 16, respectively. After clustering the samples by *k*-means algorithm, the center *c*_*i*_ of each hidden unit is determined. The activation function of the RBF neural networks is determined to be radial basis function. For the MLP and RBF networks, the hyperparameters are adjusted to reduce the generalization error below the threshold *ε*.

Finally, outputs of each SVM group and all MLP and RBF networks are connected into one vector, which will be the input of the secondary learner. By observing the tumor marker level in the dataset, it is found that for benign tumors and patients in stage I, tumor marker levels are usually close to the normal range. But for patients in stage III and stage IV, the level of tumor markers deviates greatly from the normal range. Therefore, we assume that the growth of tumor markers in the development process of PCa conforms to the exponential law. This hypothesis is basically true in medicine. In the early stage, symptoms are very slight or not obvious. Tumors tend to be hard to find and grow slowly. However, in the middle and late stages, they grow savagely and spread throughout the body, making tumor marker levels really high.

Therefore, ELR is selected as the secondary learner to ensemble results of the SVM, MLP, and RBF models. Supervising output values 3, 4, 5, and 6 are added manually for input patient samples in stages I, II, III, and IV, respectively. What may be wired is that the output value of ELR is not set to start from 1. It is considered for the reason of improving the model's robustness to normal people and benign tumor cases. Finally, the evaluation value of PCa's malignancy (EM value) is output.  Algorithm 1 shows the procedure that integrates the results of base learners by ELR.

The system determines the stage of malignant PCa according to the EM value and recommends the appropriate treatment method. Commonly used tumor treatment methods include chemotherapy, radiotherapy, excision, drug method, and hospital charge. After treatment methods are recommended by the system, doctors will decide to take it or abandon it or make modification based on it. What must be emphasized is that in the aspect of therapy recommendation, the system mainly gives a rough strategy to relieve doctors' pressure to some extent instead of replacing doctors completely. Concrete treatment is relevant to many factors which cannot be simply given by the system. If one patient has been treated for a while, the system will track the changes of his or her tumor marker levels, input the results of the tumor marker test into the system, and output the EM value to evaluate effect of the treatment. If the EM value changes a little, the treatment plan will be changed. If the EM value decreases greatly, this means it really works, so the original plan will be maintained. During this process, every effective therapy and corresponding EM value will be recorded in the database for further use.

## 4. Experiment

### 4.1. Dataset and Models' Training

We collected a large amount of data from three top-class hospitals in China: First Xiangya Hospital, Second Xiangya Hospital, and Third Xiangya Hospital. Relevant information about the data is shown in Table 2.

After screening and preprocessing the data, relevant records of the tumor markers (PSA, PSMA, tPSA, RBC, HB, and PAP) and diagnostic results (benign, stage I, stage II, stage III, and stage IV) are obtained.

Table 3 shows the normal range of six tumor markers related to PCa. Values of malignant patients' tumor marker are several times or even tens of times beyond the normal range.

**Figure 4:**
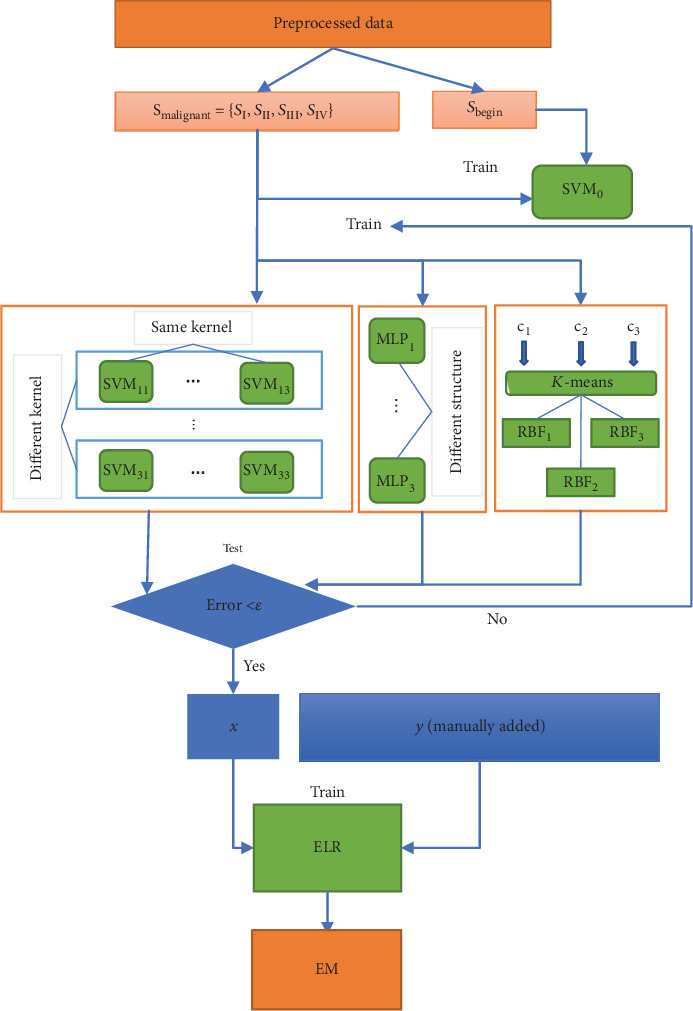
Training process of the proposed system.

Figure 4 shows the training process. The datasets are divided into two parts: training set and test set, accounting for 80% and 20%, respectively. Each of them is then divided into *S*_malignant_ and *S*_benign_. First of all, *S*_malignant_ and *S*_benign_ are used and the appropriate kernel function and penalty parameter are searched to train SVM_0_ until the test error is below *ε*. Second, malignant samples are divided into four parts *S*_malignant_ = {*S*_I_, *S*_II_, *S*_III_, *S*_IV_} according to their clinical stages. SVM and neural networks are not sensitive to data. What is more, arbitrary division of data is likely to lead to the problem of imbalanced data which means two datasets do not have the same distribution. Hence, the whole training set is used to train all base learners instead of dividing it into several parts. Each binary SVM is trained separately. Majority voting is used to ensemble the output results of SVMs in the same group. For neural networks, the malignant samples are directly marked as (1, 0, 0, 0), (0, 1, 0, 0), (0, 0, 1, 0), (0, 0, 0, 1) by their stages. What needs to be emphasized is that while training RBF neural networks, the *k*-means clustering algorithm is performed to determine the centers of hidden layers. *c*_*i*_ in Figure 4 is a hyperparameter that needs to be tuned. Back propagation and gradient descent are performed to obtain good classification ability. Finally, the output of SVM groups and neural networks are reshaped into one vector, which is used as the input of the exponential linear regression model. Artificial labels *y* are added to train the ELR model. The loss function of ELR is selected as mean square loss, namely,

(5) $$\begin{array}{c} L\left(w,b\right)=\frac{1}{m}\overset{m}{\underset{i=1}{\sum }}{\left(\text{E}{\text{M}}_{i}-\text{E}{\text{M}}_{i}^{\prime }\right)}^{2}, \end{array}$$

where EM_*i*_ is the evaluation of the *i*th patient' tumor malignance and EM_*i*_′ is the manually set supervising value.

### 4.2. Analysis of the Results of Experiments

After the model was trained, all the malignant examples in different stages were input into the model and the range of their EM values was calculated, which are listed in Table 4. From Table 4, it can be known that the EM values of all malignant examples have a rough 0.5 deviation around the supervising value set in advance. The model has good fitting ability on malignant samples of different stages, which indirectly proves our hypothesis that the tumor marker level increases exponentially with the development of tumor is credible.

**Figure 5:**
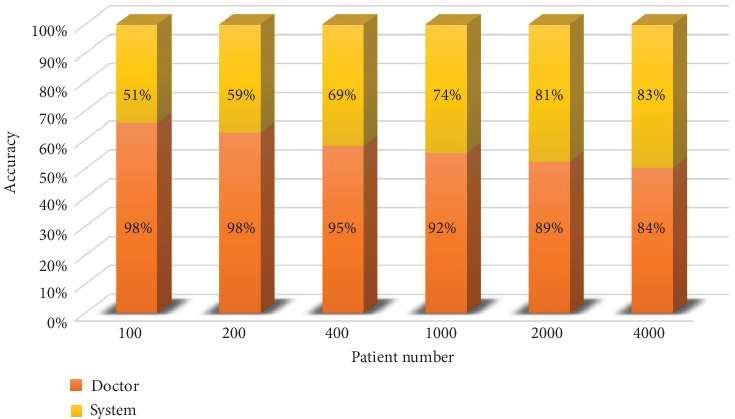
Comparison of the doctor and the system.

To verify the effectiveness of our medical decision system, we compared the accuracy of the model on different scale datasets with the accuracy of doctors. As shown in Figure 5, when the amount of data is small, the accuracy of the auxiliary medical decision system is very low, close to 50%. In this circumstance, the accuracy of doctors is really high, almost 100%. However, as the amount of data increases, the accuracy of the medical decision system increases as well. Simultaneously, doctors' accuracy starts to decline because of the burden and cumulative errors. When the amount of data reaches 4000, the accuracy of the system is roughly the same with that of doctors. This indicates that our auxiliary diagnostic system can make use of the increasing amount of data to improve generalization performance.

**Figure 6:**
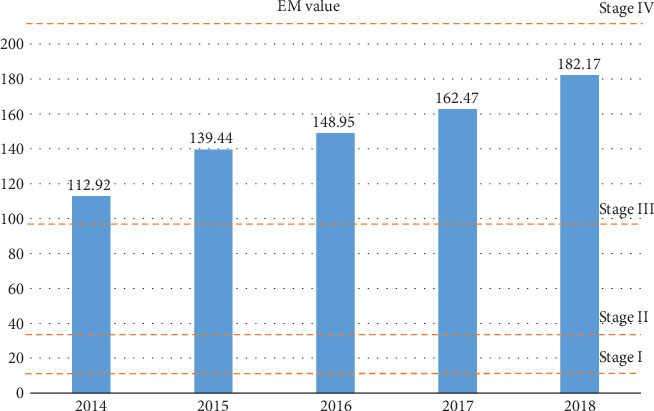
Average EM value in the past five years.

We also calculated the average EM value of different years to explore the development trend of PCa in recent years. As shown in Figure 6, the mean EM value of patients from three hospitals has been gradually increasing since 2014. This implies an increase in the number or proportion of patients with malignant prostate cancer which will make medical resources scarcer, so it is necessary and urgent to establish an auxiliary medical decision system based on big data.

**Figure 7:**
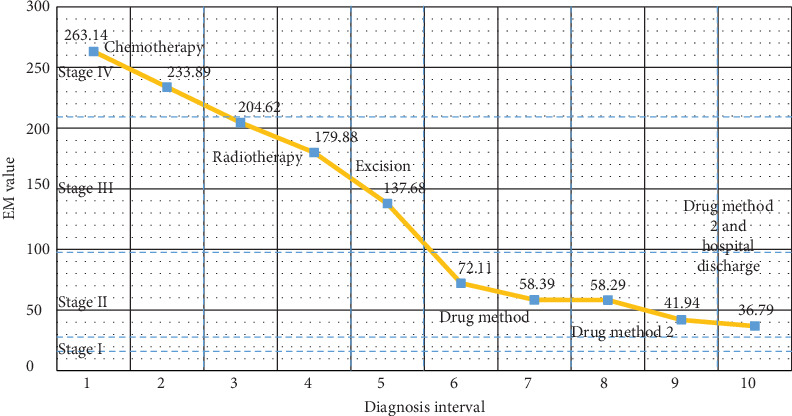
A typical treatment process of a PCa patient.

Because our medical decision system can quantitatively evaluate the malignancy of prostate cancer, it can easily judge the efficacy of the treatment plan by its EM value change and recommend treatment methods to improve the condition of PCa patients according to their EM levels.  Figure 7 shows the recommended treatment methods and changes of the EM value of a patient whose EM value is very high at first. In the end of the diagnosis interval, the patient's EM value is relatively low, which proves the tumor has been controlled by the recommended treatment plan. It can be concluded that the treatment methods recommended by the system can effectively improve the condition of cancer patients and prolong the survival time for patients in stage III or IV.

### 4.3. Relevant Analysis Based on the System

**Figure 8:**
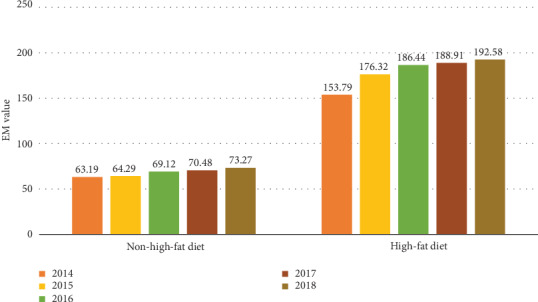
Contrast of people with different diet habits.

Since our medical decision system can evaluate the malignancy of tumors, by controlling different input variables, the influence of a certain factor on prostate cancer can be effectively evaluated. Here, relevant information of some patients was collated. Then, influence of patients' diet habits and genetic inheritance on prostate cancer was evaluated. Diet habits are mainly divided into high-fat diets and non-high-fat diets according to the description in the patient's medical history. From the data of 2014-2018, it can be seen that the condition of patients with high-fat diet tends to be more severe. The EM value for patients with high-fat diet is in the range of 150-190, while for those with non-high-fat diet, EM value is only 60-70, as shown in Figure 8.

**Figure 9:**
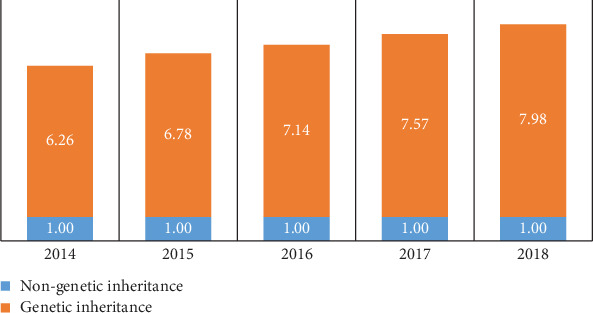
Contrast of people with or without genetic inheritance.

Genetic inheritance is defined by a cancer case in the patient's family members. The results show that patients with genetic inheritance have a cancer malignancy that is 6 to 7 times that of patients without genetic inheritance, which can be seen in Figure 9.

## 5. Conclusion

This paper mainly builds an auxiliary medical decision system of PCa for developing countries that lack medical resources. The system is able to provide doctors with advice on the diagnosis, staging, and treatment method of prostate cancer. After training the system in big data environment, although its accuracy continues to rise, it still cannot replace professional doctors and can only be used as an auxiliary diagnostic system to relieve the burden of doctors. Based on this system, we have researched the development of prostate cancer in the past five years and found that the prevalence of prostate cancer is increasing. In addition, high-fat diet and genetic inheritance increase the severity of the disease. The next stage of this research will consider introducing other medical detection information, such as CT, MRI, and PET-CT, to further improve the accuracy and credibility of the system.

## Figures and Tables

**Algorithm 1:**
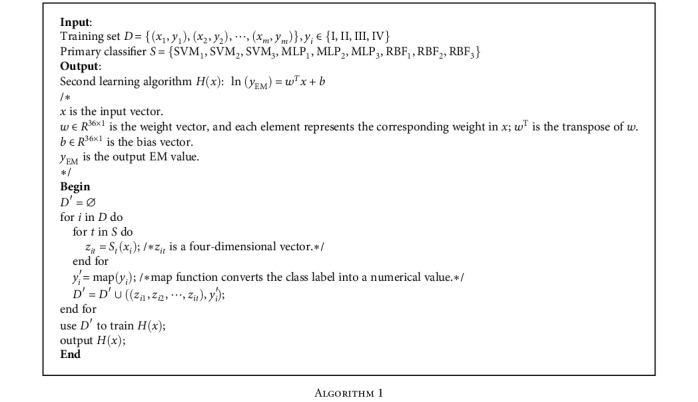


**Table 1:** Commonly used kernel functions.

Kernel functions	Formula
Linear kernel	*κ*(*x*_*i*_, *x*_*j*_) = *x*_*i*_^T^*x*_*j*_
Polynomial kernel	*κ*(*x*_*i*_, *x*_*j*_) = (*x*_*i*_^T^*x*_*j*_)^*d*^
Gauss kernel	$\kappa \left(x_{i},x_{j}\right)=\exp\left(-\frac{{\left\|x_{i}-x_{j}\right\|}^{2}}{2\sigma^{2}}\right)$
Sigmoid kernel	$\kappa \left(x_{i},x_{j}\right)=\exp\left(-\frac{\left\|x_{i}-x_{j}\right\|}{\sigma }\right)$
Laplace kernel	*κ*(*x*_*i*_, *x*_*j*_) = tanh(*βx*_*i*_^T^*x*_*j*_ + *θ*)

**Table 2:** Type and number of collected data.

Data type	Number
Patient information	1,933,535 items
Outpatient service	691,238 people
Doctors' device in outpatient	24,021,298 items
Be hospitalized	1,149,187 people
Diagnosis	1,089,327 items
Electronic medical records	4,855,619 items
Doctors' device in clinical	25,757,699 items
Inspection records	157,426 items
Medical laboratory records	8,725,586 items
Routine inspection records	22,358,881 items
Operation records	318,022 items
Drug records	120,546 items

**Table 3:** Normal range of different tumor markers.

Types of tumor marker	Normal range
Prostate-specific antigen	0-4.0 ng/mL
Total prostate-specific antigen	4-20 *μ*g/L
Hemoglobin	120-165 g/L
Red blood cell	12-15 g/100 mL
Prostate acid phosphatase	0-9 U/L
Prostate-specific membrane antigen	0-4 ng/mL

**Table 4:** EM value of each stage of PCa.

Clinical stage of PCa	Range of lnEM
Stage I	2.7-3.6
Stage II	3.6-4.5
Stage III	4.5-5.3
Stage IV	>5.3

## Data Availability

All medical data management and system come from Central South University. If readers are interested in those data, you can visit http://www.xiangya.com.cn/english/. All data analyzed during the current study are included in the submission.
